# Interactions Between the Gut Microbiome and Genetic and Clinical Risk Factors for Metabolic Dysfunction-Associated Steatotic Liver Disease (MASLD) in Patients with Type 2 Diabetes Mellitus from Different Geographical Regions of Argentina

**DOI:** 10.3390/life16020283

**Published:** 2026-02-06

**Authors:** Bárbara Suarez, Adriana Mabel Álvarez, María Florencia Mascardi, Ana Laura Manzano Ramos, Dong Hoon Woo, María Mercedes Gutiérrez, Guillermo Alzueta, María del Carmen Basbus, Santiago Bruzone, Patricia Cuart, Guillermo Dieuzeide, Teresita García, Olga Escobar, Ramón Diego José Carulla, Cristina Oviedo, Natalia Segura, Olguita Del Valle Vera, Javier Nicolás Giunta, Adrián Gadano, Julieta Trinks

**Affiliations:** 1 Instituto de Medicina Traslacional e Ingeniería Biomédica (IMTIB), Consejo Nacional de Investigaciones Científicas y Técnicas (CONICET), Universidad del Hospital Italiano (UHIBA), Hospital Italiano de Buenos Aires (HIBA), Buenos Aires C1199ACJ, Argentina; barbara.suarez@hospitalitaliano.org.ar (B.S.); maria.mascardi@hospitalitaliano.org.ar (M.F.M.); 2 Consejo Nacional de Investigaciones Científicas y Técnicas (CONICET), Buenos Aires C1425FQB, Argentina; 3 Endocrinology Unit, Department of General Medicine, Hospital Italiano de Buenos Aires (HIBA), Buenos Aires C1199ABB, Argentina; adriana.alvarez@hospitalitaliano.org.ar (A.M.Á.); ana.manzano@hospitalitaliano.org.ar (A.L.M.R.); javier.giunta@hospitalitaliano.org.ar (J.N.G.); 4 Nutrition Unit, Department of General Medicine, Hospital Italiano de Buenos Aires (HIBA), Buenos Aires C1199ABB, Argentina; dong.woo@hospitalitaliano.org.ar (D.H.W.); mercedes.gutierrez@hospitalitaliano.org.ar (M.M.G.); 5 Independent Researcher, Balcarce B7620ELO, Buenos Aires Province, Argentina; g_alzueta@yahoo.com.ar; 6 Centro de Investigaciones Endocrinológicas (CIDEN) Private Center, San Salvador de Jujuy Y4600APP, Jujuy Province, Argentina; maria_basbus@yahoo.com.ar; 7 Instituto de Diagnóstico e Investigaciones Metabólicas (IDIM) Private Center, Buenos Aires C1406GLN, Argentina; sanbruzone@hotmail.com; 8 Diagnostic Center, Ituzaingó W3302, Corrientes Province, Argentina; drapatriciacuart@gmail.com; 9 Center for Integral Care in Diabetes, Chacabuco B6740ELF, Buenos Aires Province, Argentina; dieuzeideg@gmail.com; 10 Centro Médico de Atención y Prevención Clínica (CMIC) Private Center, San Miguel de Tucumán T4000EBC, Tucumán Province, Argentina; tngarciag@gmail.com; 11 Dr. Mario Stivala Public Hospital, La Cocha T4164, Tucumán Province, Argentina; draolgaescobar@gmail.com; 12 Albarracín Medical Consultation Center, Bariloche R8400BFC, Río Negro Province, Argentina; diegocarulla@hotmail.com.ar; 13 Independent Researcher, Buenos Aires C1405CDT, Argentina; crisvivi@hotmail.com.ar; 14 Dr. Alberto Maggio Municipal Diabetes Center, Malvinas Argentinas B1613FIJ, Buenos Aires Province, Argentina; nsegpsn@gmail.com; 15 Independent Researcher, Río Gallegos Z9400BAM, Santa Cruz Province, Argentina; olguitavera18@gmail.com; 16 Hepatology Unit, Department of General Medicine, Hospital Italiano de Buenos Aires (HIBA), Buenos Aires C1199ABB, Argentina; adrian.gadano@hospitalitaliano.org.ar

**Keywords:** MASLD, microbiota, diabetes: FIB-4, PNPLA3

## Abstract

**Background:** Local specific biomarkers for MASLD risk stratification are urgently needed in Argentina. **Aim:** The aim of the study was to characterize the interaction of gut microbiome signatures and genetic and clinical risk factors for MASLD in patients with diabetes from different regions of Argentina. **Materials and Methods:** We recruited 214 patients with diabetes from different regions of Argentina. Anthropometric, clinical, and lifestyle data were obtained from all participants, who also underwent abdominal ultrasound for MASLD diagnosis and oral swabbing. The *PNPLA3* gene was amplified by PCR from the swabs, and the rs738409 genotype was determined via bidirectional sequencing. To profile the MASLD-associated microbiome, stool was collected from 170 participants. V4 16S rRNA gene sequencing was performed, and reads were analyzed using QIIME2 2024.10.1. R Studio 2023.05.1 was used for statistical analyses. **Results:** MASLD prevalence was 77.9%, with similar rates of occurrence in all regions represented. FIB-4 scores < 1.3 and > 2.67 were detected in 55.3% and 7.4% of patients, respectively. Half of the diabetic patients had the *PNPLA3* GG genotype, with the highest rates occurring in patients from Northwestern Argentina (64.9%; *p* = 0.02 vs. Buenos Aires). The *PNPLA3* GG genotype was an independent risk factor for FIB-4 score (*p* = 0.0008) and a protective factor against glycated hemoglobin (*p* = 0.004), fasting plasma glucose (*p* = 0.008), and cholesterol levels (*p* = 0.02). Marked regional differences were observed in microbiota diversity and composition in Argentina. After adjusting for geographical region, *Negativibacillus* genus was exclusively detected in diabetic patients with MASLD and GG carriers. The *Catenibacterium* genus was related to FIB-4 > 2.67. Short-chain fatty acid-producing bacteria were linked to the absence of MASLD. **Conclusions:** Although some geographical regions of Argentina were not represented in this study and these results therefore cannot be generalized to the country as a whole, these specific signatures could be useful as biomarkers for MASLD risk stratification in Argentines with diabetes.

## 1. Introduction

Measures intended to overcome the rising epidemic of metabolic dysfunction-associated steatotic liver disease (MASLD) have centered on identifying people at the highest risk of disease progression, so that they can be offered timely interventions [1]. Due to the limitations of liver biopsy, the need for new risk stratification methods has prompted the search for non-invasive diagnostics for MASLD, especially for steatohepatitis and fibrosis. These new methods mostly depend on biomarkers and scores derived from clinical and biochemical data, imaging techniques, and microbiome signatures [2,3]. However, despite best efforts, these methods cannot yet be considered analytically valid tests that are useful in MASLD clinical practice [4].

Another obstacle to achieving an MASLD risk categorization that is inclusive of worldwide populations is the ongoing challenge of accurately assessing the disease prevalence and its burden. While the global prevalence of MASLD is estimated to be 30.2%, there is a lack of high-quality data for specific regions and populations [5,6]. This scenario is worrisome in Latin America, where the prevalence of MASLD is the highest in the world (44.4%) [7]. Inequities in the region influence access to MASLD diagnosis and screening, resulting in a lack of representative data [6,8].

Moreover, a population’s ethnic background significantly impacts the performance of MASLD risk stratification tools. For example, the FIB-4 index, a non-invasive test for assessing liver fibrosis, may underperform in Black individuals [9]. In addition, lower cut-points for elastography techniques may be needed to optimize surveillance for significant fibrosis in Latin American patients with MASLD [10]. Furthermore, the rs738409 (c.444C > G) polymorphism in the patatin-like phospholipase domain-containing 3 (*PNPLA3)* gene, which encodes the I148M variant associated with an individual’s susceptibility to MASLD and disease severity, exhibits the strongest effect and the highest prevalence in Latin America [11]. Finally, to date, although no specific gut microbiota signature has been reliably connected to any particular geographic area or ethnic group [12], alterations in bacterial composition and metabolic functions may affect systemic inflammation and induce changes in liver fat that differ across ethnic groups [13].

In this regard, Latin America with its multi-ethnic population [14] poses an extra challenge for MASLD risk stratification that reinforces the need for local studies with large cohorts, that suitably depict the intrinsic interindividual diversity and regional and demographic variations in MASLD studies [15]. Thus, this study aimed to determine the prevalence of MASLD and analyze the interaction of gut microbiome signatures and genetic and clinical risk factors for MASLD in patients with type 2 diabetes mellitus (T2DM) from different geographical areas of Argentina.

## 2. Materials and Methods

### 2.1. Study Population

In 2023–2024, a cohort of 250 unrelated patients in outpatient care diagnosed with T2DM who had participated in a MASLD prevalence study in Argentina [16] were recruited. In order to account for the sample’s representativeness of all environments and populations of Argentina, endocrinologists recruited participants from 12 diabetes centers from different regions of the country (Figure S1). The regions were as follows: urban area of Buenos Aires city (BA city, *n* = 82), rural area of Buenos Aires province (rural BA, *n* = 50), northeastern Argentina (NEA, *n* = 25), northwestern Argentina (NWA, *n* = 58), and “Patagonia” or southern Argentina (SOUTH, *n* = 35). Individuals with schistosomiasis or any liver disease other than MASLD, complications of end-stage liver disease, human immunodeficiency virus (HIV) infection, hepatitis B virus (HBV) infection, hepatitis C virus (HCV) infection, dietary restrictions (e.g., gluten-free, ketogenic, vegetarian or vegan), and/or significant alcohol consumption (>30 g/day for males and >20 g/day for females) were excluded from the study. Other exclusion criteria comprised the following: subjects who used recreational drugs or who had been prescribed medications known to cause elevations in liver transaminases; those who used antibiotics, laxatives or probiotics in the 6 months before the study; those with a history of pelvic radiation exposure or chemotherapy; those having undergone previous gastrointestinal surgery modifying their anatomy; and those with a history of chronic gastrointestinal disease, inflammatory bowel disease, or any other gut infectious disease. Pregnant women and those who lactate were also not invited to participate. In total, 36 out of the 250 initially recruited-participants (14.4%) met one of these criteria (Figure S2) and were hence excluded from the study. A total of 214 patients were thus considered eligible to participate (Figure S2).

The study protocol was developed in accordance with the Declaration of Helsinki. Approval was obtained from the Ethics Committee of Hospital Italiano de Buenos Aires. All study participants signed a written informed consent.

### 2.2. Data and Sample Collection

Demographic and anthropometric data (height, weight and body mass index or BMI) and medical history (hypertension, cardiovascular disease history, physical activity, alcohol consumption and medications) were obtained from each subject.

At the time of recruitment, 172 patients (90.5%) were receiving diabetes treatment. Metformin (47.3%) was the most common glucose-lowering oral agent prescribed, followed by dipeptidyl peptidase 4 (DPP4) inhibitors (12.8%), sodium glucose transport protein 2 (SGLT2) inhibitors (11.6%), and glucagon-like peptide-1 (GLP-1) agonists (8.3%). Treatment with DPP4 inhibitors was significantly lower in rural BA (*q* = 0.04), whereas GLP-1 agonists were more commonly prescribed in urban and rural BA (*q* = 0.01).

Standardized laboratory methods were used to measure fasting glucose, glycated hemoglobin (HbA1c), platelet count, total cholesterol, triglycerides, aspartate aminotransferase (AST), and alanine aminotransferase (ALT) levels.

Cardiovascular risk was assessed using the calculator from the “HEARTS in the Americas” initiative developed by the Pan American Health Organization (PAHO) and the World Health Organization (WHO). A systolic blood pressure ≥ 130 mmHg, a diastolic blood pressure ≥ 85 mmHg, or taking medication for controlling high blood pressure was considered as having hypertension. Physical activity was self-reported by all subjects.

MASLD diagnosis was carried out by ultrasound, following standard medical practice. The risk of advanced liver fibrosis was determined via the FIB-4 index. FIB-4 < 1.3 was considered low risk, scores between 1.3 and 2.67 were in the indeterminate risk range, and FIB-4 > 2.67 was considered high risk.

Moreover, participants were requested to perform a sterile buccal swab and to collect approximately 5 g of stool in a sterile bacteriostatic buffer tube, with the stool including parts from the entire bowel movement. Both samples were kept at room temperature and sent within 14 days to our laboratory, where they were stored upon arrival at −20 °C until further analysis. Forty-four (20.6%) participants did not provide a stool sample (Figure S2).

### 2.3. Isolation of Human Genomic DNA and Determination of PNPLA3 rs738409 Genotype

Genomic DNA was isolated from buccal swabs using QIAamp DNA Blood Mini Kit (QIAGEN, Hilden, Germany) according to the manufacturer’s protocol. A 668-bp fragment of the *PNPLA3* gene was amplified as previously described [17]. The primers used in the reaction were 5′-CGA TCT AGC CCC TTT CAG TC-3′ (forward) and 5′-GCA GAT TAA GTG AAC CAG CC-3′ (reverse). The PCR conditions were: 30 cycles of denaturation for 30 s at 94 °C, annealing for 30 s at 62 °C, and extension for 1 min at 72 °C. The presence of the rs738409 genotype (CC, CG, or GG) was confirmed by bidirectional sequencing with Big-Dye Termination chemistry system using the ABI Prism sequence detection system ABI3730 (Applied Biosystems, Life Technologies Corp., Foster City, CA, USA). The sequencing chromatogram was examined using BioEdit Sequence Alignment Editor version 7.1.3.0.

### 2.4. Microbial DNA Extraction, 16S rRNA Library Preparation and NGS

Fecal DNA was isolated from each sample using QIAamp DNA Stool Mini Kit (QIAGEN^®^) following the manufacturer’s instructions.

Library preparation was carried out following the procedures described in the Earth Microbiome Project (EMP) 16S Illumina Amplicon library preparation methodology (http://www.earthmicrobiome.ucsd.edu), with Illumina 16S V4 primer constructs 515F (Parada)-806R (Apprill) [18,19]. A total of 170 samples were sequenced using Illumina^®^ HiSeq 3000 (San Diego, CA, USA) for 2 × 150-base pair (bp) reads along with a 10 bp index region.

### 2.5. Bioinformatic Processing and Statistical Analysis

Reads were processed using QIIME2 (version 2024.10.1) [20]. Reads were trimmed, merged, and denoised, and representative sequences were chosen using the Deblur2 plugin [21].

Qiime fragment-insertion SEPP (version 2024.10.0) was used to set each sequence into a reference phylogenetic tree (sepp-refs-gg-13-8.qza reference database) [22]. The QIIME2 feature classifier, utilizing the BLAST+ algorithm version 2.14.0 [23], aligned taxa against Greengenes2 2022.10 from the 515F/806R region of sequences [24].

Samples were reduced in QIIME2 to the same number of reads (sequencing depth) by randomly removing sequences from deeper samples, using the sample with the fewest QC-passed sequences (328,244) as the benchmark to ensure a fair comparison of diversity metrics (like species richness) across different samples. Shannon index alpha diversity was calculated, and the Kruskal–Wallis test was used to determine its significance. On the other hand, the significance of differences in beta diversity between groups was calculated with the adonis function of the vegan R package via a PERMANOVA analysis of Bray–Curtis distances [25].

To determine differences in taxa abundance at the family, genus, and species levels, microbiome compositional analysis with a bias correction 2 (ANCOMBC2) framework [26] in R Studio (2023.05.1) was. Core microbiome from the R microbiome package [27] was used to calculate the group of sequence variants at the genus level detected in 50–100% of the samples with a relative abundance threshold value above 0.001% (core microbiota).

Given that the regional factor had the highest effect on the microbiome composition (R^2^ = 0.03), the PERMANOVA and ANCOMBC2 analyses for MASLD diagnosis, the FIB-4 score and the *PNPLA3* rs738409 genotype were performed after adjusting for the geographical origin of the samples as a confounder.

Data were presented either as a direct visualization of QIIME2 artifacts on QIIME2 View, or using ggplot2 [28], with data from R Studio (2023.05.1) or data extracted from QIIME2 artifacts using qiime2R (version 0.99.20; https://github.com/jbisanz/qiime2R accessed on 29 December 2025).

### 2.6. Statistical Analysis

Data for continuous variables are shown as medians and interquartile ranges (IQRs), which represent the distance between the first quartile (Q1) and the third quartile (Q3). Categorical variables are presented as proportions (%). Statistical analyses were carried out using R (version 4.0.5). Chi-Square and Kruskal–Wallis ANOVA tests were used to establish differences between groups for categorical and continuous metadata, respectively. Post hoc Dunn *p* values with Benjamini–Hochberg FDR adjustment (*q* values) were evaluated.

The associations between the *PNPLA3* rs738409 genotype and demographical data, geographical origin and clinical variables were assessed using Spearman’s correlation analysis and logistic regression analysis. Univariate and multivariate logistic analyses were performed to identify variables that were independently associated with the *PNPLA3* genotype. All significant parameters in the univariate analysis were included in the multivariate analysis, which was adjusted for age, gender, and BMI.

## 3. Results

### 3.1. Demographic, Clinical and PNPLA3 Genetic Background of the Study Subjects

Gender, age and BMI distribution were similar across all regions (Table 1). However, waist circumference was significantly higher in BA city (*q* = 0.006), and the lowest rates of physical activity were observed in rural BA (*q* = 0.005); meanwhile, high blood pressure and cardiovascular risk score were significantly higher in the latter region and in NEA (*q* = 0.03 and *q* = 0.02, respectively).

MASLD was diagnosed with the help of abdominal ultrasound in 77.9% of T2DM patients, and no significant differences were observed between regions (Table 1). FIB-4 scores > 2.64 were more frequently observed in rural BA (*q* = 0.01; Table 1). Cardiometabolic comorbidities were present in patients with FIB-4 scores > 2.64, as follows: obesity (62%), hypertension (80.9%) and dyslipidemia (54%). The median duration of diabetes in this group of patients was 11 (5–15.5) years. When compared to patients with low or intermediate risk of fibrosis, differences were not statistically significant in all cases (*p* < 0.05).

The *PNPLA3* gene was successfully amplified in 190 samples (62 from BA city, 40 from rural BA, 37 from NWA, 20 from NEA, and 31 from SOUTH). Half of them displayed the GG genotype, with the highest prevalence found in NWA (64.9%) and NEA (60%) and the lowest in BA city (40.3%; *p* = 0.02 vs. NWA).

### 3.2. Correlations Between PNPLA3 rs738409 Genotype and Clinical Markers

Logistic regression analysis revealed that the GG genotype was considered a risk factor independently associated with FIB-4 scores (OR = 2.06; *p* = 0.0008) and a protective factor against HbA1c (OR = 0.875; *p* = 0.004), fasting plasma glucose (OR = 0.73; *p* = 0.008), and cholesterol (OR = 0.525; *p* = 0.02) levels (Table 2 and Figure S3).

### 3.3. Analyses of the Gut Bacterial Metagenome of T2DM Patients

After sequencing the hypervariable V4 region of the bacterial 16S gene, 25 out of the 170 samples were excluded due to their low number of reads, which resulted in the inclusion of 145 samples (60 from BA city, 40 from rural BA, 35 from NWA, and 10 from SOUTH). The median depth of sequencing, after the exclusion of low-depth libraries, was 774,721 reads per sample (IQR: 345,683 reads). Rarefaction plots showed that the sequence depth was adequate to capture the bacterial community richness and diversity (Figure S4).

#### 3.3.1. Analyses of the Gut Bacterial Metagenome According to the Geographical Origin of the Samples

**Figure 1:**
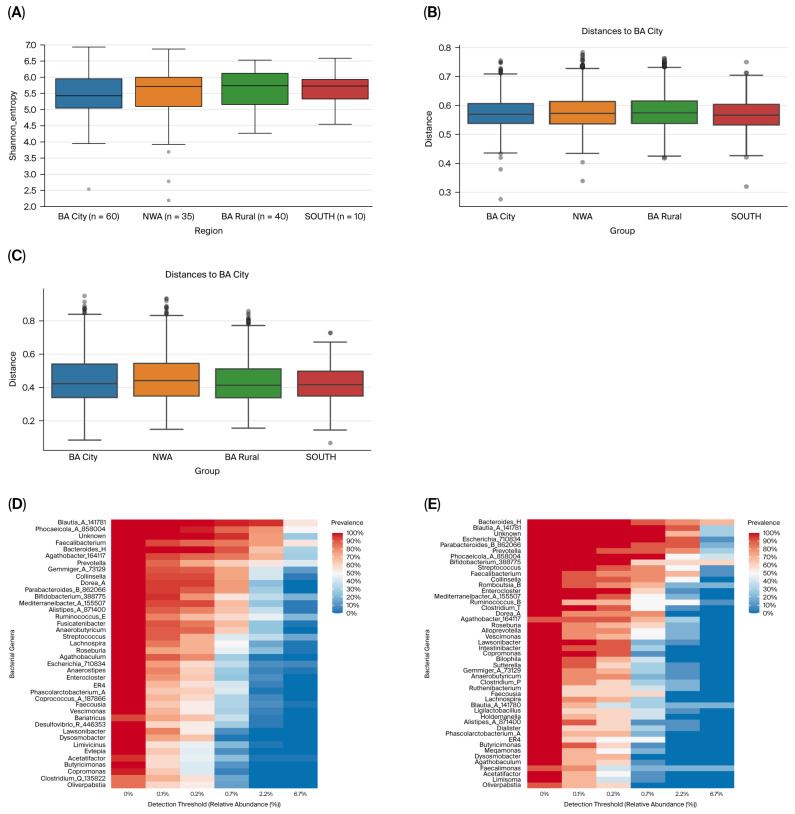

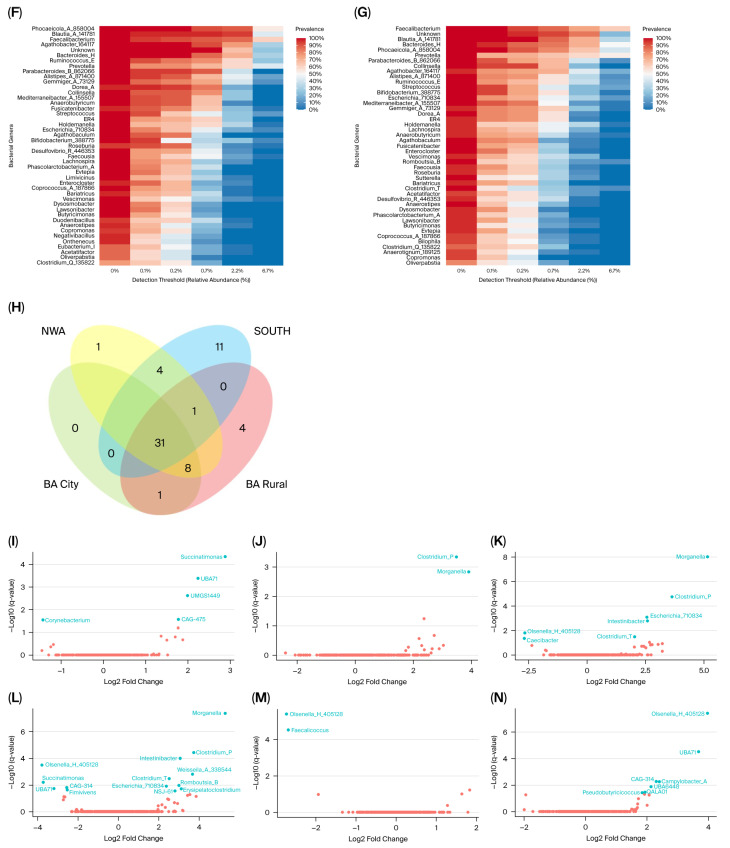
Bacterial diversity and taxa abundance differences in the gut microbiota of T2DM patients from various geographical regions in Argentina. (**A**) Shannon diversity index plotted based on the geographical origin of the samples. (**B**) Unweighted UniFrac distances (beta diversity) plotted based on the geographical region of origin of the samples. (**C**) Weighted UniFrac distances (beta diversity) plotted based on the geographical region of origin of the samples. (**D**) Core microbiome for samples from BA city. (**E**) Core microbiome for samples from SOUTH. (**F**) Core microbiome for samples from rural BA. (**G**) Core microbiome for samples from NWA. (**H**) Venn diagrams represent shared core genera between groups. (**I**) Volcano plot from ANCOMBC2 analysis at the genus level between BA city and rural BA. (**J**) Volcano plot from ANCOMBC2 analysis at the genus level between NWA and SOUTH. (**K**) Volcano plot from ANCOMBC2 analysis at the genus level between BA city and SOUTH. (**L**) Volcano plot from ANCOMBC2 analysis at the genus level between rural BA and SOUTH. (**M**) Volcano plot from ANCOMBC2 analysis at the genus level between BA city and NWA. (**N**) Volcano plot from ANCOMBC2 analysis at the genus level between NWA and rural BA. In each volcano plot, the *x*-axis (effect size) shows the log2 fold change, which represents the magnitude of the difference between the two analyzed geographical regions. Negative values indicate features that are more abundant in the first- mentioned region in the plot legend, while positive values on the *x*-axis indicate that features are more abundant in the second-mentioned geographical region. The *y*-axis (significance) shows the −log10 (*q* values). A larger negative log-transformed *q* value means stronger statistical significance. The threshold for significance was set as *q* < 0.05, i.e., −log10 (FDR *q* value) > 1.3. ANCOMBC2, Analysis of Composition of Microbiomes with Bias Correction 2.

Samples from SOUTH and rural BA had the lowest Shannon entropy indexes while those from BA city and NWA showed the highest levels of alpha diversity, with no significant differences across geographical regions (*p* = 0.54; Figure 1A).

Beta diversity (considering weighted and unweighted UniFrac distances) revealed that samples from SOUTH showed a statistically significant separation from those of the remaining regions in the unweighted (*q* = 0.003; Figure 1B) and weighted (*q* = 0.031; Figure 1C) Unifrac plots.

Core microbiota for samples from BA city comprised 40 bacterial genera (corresponding to 6.94% of the genera present in the group; Figure 1D), whereas the core microbiota in SOUTH consisted of 47 genera (9.77% of the total, Figure 1E), and 45 genera in both rural BA (8.74% of the total; Figure 1F) and in NWA (8.23% of the total; Figure 1G). No bacterial genera were exclusively observed in BA city (Figure 1H). However, four genera of the core microbiota were considered exclusive to rural BA (g__*Onthenecus*, g__*Eubacterium_I*, g__*Negativibacillus*, and g__*Duodenibacillus*), one genus (g__*Anaerotignum*_189125) to NWA, and eleven genera (g__*Alloprevotella*, g__*Limisoma*, g__*Clostridium_P*, g__*Blautia_A_141780*, g__*Faecalimonas*, g__*Ruminococcus*_B, g__*Ruthenibacterium*, g__*Intestinibacter*, g__*Megamonas*, g__*Dialister*, and g__*Ligilactobacillus*) to SOUTH (Figure 1D–H).

Differentially abundant taxa were identified between geographical regions (Figure 1I–N and Figure S5A–K). Rural BA differentiated itself from BA city by a higher abundance of *Succinatimonas* (*q* = 4.78 × 10^−5^), *UBA71* (*q* = 0.0004), *UMGS1449* (*q* = 0.002), and *CAG-475* (*q* = 0.03) bacterial genera, whereas the *Corynebacterium* genus (*q* = 0.03) prevailed in BA city (Figure 1I). Moreover, SOUTH displayed the highest number of differential abundant taxa after comparison with other geographical areas (Figure 1J–L). For instance, it showed more abundance of *Morganella* (*q* = 0.001), and *Clostridium_P* (*q* = 0.0005) genera when compared with NWA (Figure 1J); it also displayed more of these previous two bacterial genera (*q* = 9.96 × 10^−9^ and *q* = 1.84 × 10^−5^, respectively) in addition to *Escherichia_710834* (*q* = 0.0008), *Intestinibacter* (*q* = 0.002), and *Clostridium_T* (*q* = 0.03) when compared with BA city (Figure 1K); and finally, SOUTH had a higher prevalence of the aforementioned genera, *Weissella_A_338544* (*q* = 0.001), *Romboutsia_B* (*q* = 0.01), *Erysipelatoclostridium* (*q* = 0.02), and *NSJ-61* than rural BA (*q* = 0.01; Figure 1L).

#### 3.3.2. Analyses of the Gut Bacterial Metagenome According to the MASLD Diagnosis

**Figure 2:**
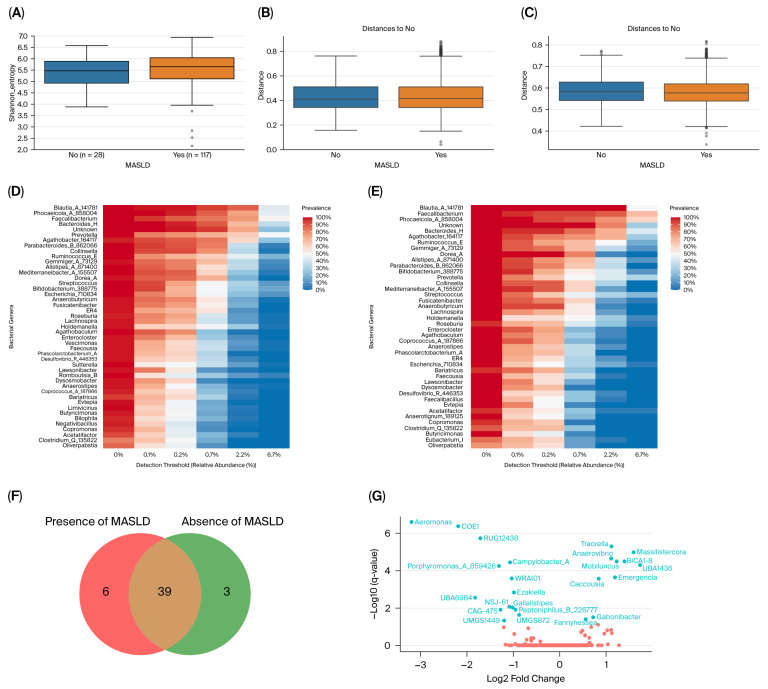
Bacterial diversity and taxa abundance differences in the gut microbiota of T2DM patients with or without MASLD. (**A**) Shannon diversity index plotted based on MASLD diagnosis. (**B**) Weighted UniFrac distances (beta diversity) plotted based on MASLD diagnosis. (**C**) Unweighted UniFrac distances (beta diversity) plotted based on MASLD diagnosis. (**D**) Core microbiome for MASLD samples. (**E**) Core microbiome for non-MASLD samples. (**F**) Venn diagrams represent shared core genera between groups. (**G**) Volcano plot from ANCOMBC2 analysis at the genus level between non-MASLD and MASLD samples. The *x*-axis (effect size) shows the log2 fold change, which represents the magnitude of the difference between the two analyzed groups. Negative values indicate features that are more abundant in the non-MASLD group, while positive values on the *x*-axis indicate that features are more abundant in the MASLD group. The *y*-axis (significance) shows the −log10 (*q* values). A larger negative log-transformed *q* value means stronger statistical significance. The threshold for significance was set as *q* < 0.05, i.e., −log10 (FDR *q* value) > 1.3. ANCOMBC2, Analysis of Composition of Microbiomes with Bias Correction 2.

Shannon entropy indices were similar between MASLD and non-MASLD samples (*p* = 0.56; Figure 2A), and no significant differences were observed in the weighted (*q* = 0.81; Figure 2B) or unweighted UniFrac distances (*q* = 0.9; Figure 2C) between both groups, after adjusting for geographical origin.

Core microbiota for the MASLD group consisted of 45 bacterial genera (7.38% of the total; Figure 2D) and 42 (7.83% of the total) for the non-MASLD (Figure 2E). Thirty-nine genera were present in the core microbiota of both groups (Figure 2F). However, there were six genera present in the MASLD group that were absent from the non-MASLD samples (g__*Bilophila*, g__*Limivicinus*, g__*Vescimonas*, g__*Negativibacillus*, g__*Romboutsia_B*, and g_*Sutterella*), and three genera (g__*Anaerotignum*_189125, g__*Eubacterium*_I, and g__*Faecalibacillus*) that were exclusively present in the non-MASLD core (Figure 2D–F).

Taxa abundance significantly differed between MASLD and non-MASLD groups (Figure 2G and Figure S6A,B). Of the 382 observed genera, the abundance of the *Traorella* (*q* = 5.28 × 10^−6^), *Massilistercora* (*q* = 1.07 × 10^−5^), *BICA1-8* (*q* = 3.33 × 10^−5^), *Mobiluncus* (*q* = 3.34 × 10^−5^), *UBA1436* (*q* = 5.19 × 10^−5^), *Anaerovibrio* (*q* = 2.34 × 10^−5^), *Emergencia* (*q* = 2.46 × 10^−4^), *Caccousia* (*q* = 2.8 × 10^−4^), *Gabonibacter* (*q* = 3.23 × 10^−2^), and *Fannyhessea* (*q* = 4.19 × 10^−2^) genera was higher among patients with MASLD (Figure 2G). On the other hand, the *Aeromonas* (*q* = 2.64 × 10^−7^), *COE1* (*q* = 4.36 × 10^−7^), *RUG12438* (*q* = 1.93 × 10^−6^), *Campylobacter_A* (*q* = 3.74 × 10^−5^), *Porphyromonas_A_859426* (*q* = 5.78 × 10^−5^), *WRAI01* (*q* = 2.75 × 10^−4^), *Ezakiella* (*q* = 1.49 × 10^−3^), *UBA6984* (*q* = 2.82 × 10^−3^), *NSJ-61* (*q* = 8.72 × 10^−3^), *Gallalistipes* (*q* = 9.72 × 10^−3^), *Peptoniphilus_B_226777* (*q* = 1.25 × 10^−2^), *CAG-475* (*q* = 1.25 × 10^−2^), *UMGS872* (*q* = 2.47 × 10^−2^), and *UMGS1449* (*q* = 4.81 × 10^−2^) genera were more abundant among those patients without MASLD (Figure 2G).

#### 3.3.3. Analyses of the Gut Bacterial Metagenome According to the FIB-4 Score

**Figure 3:**
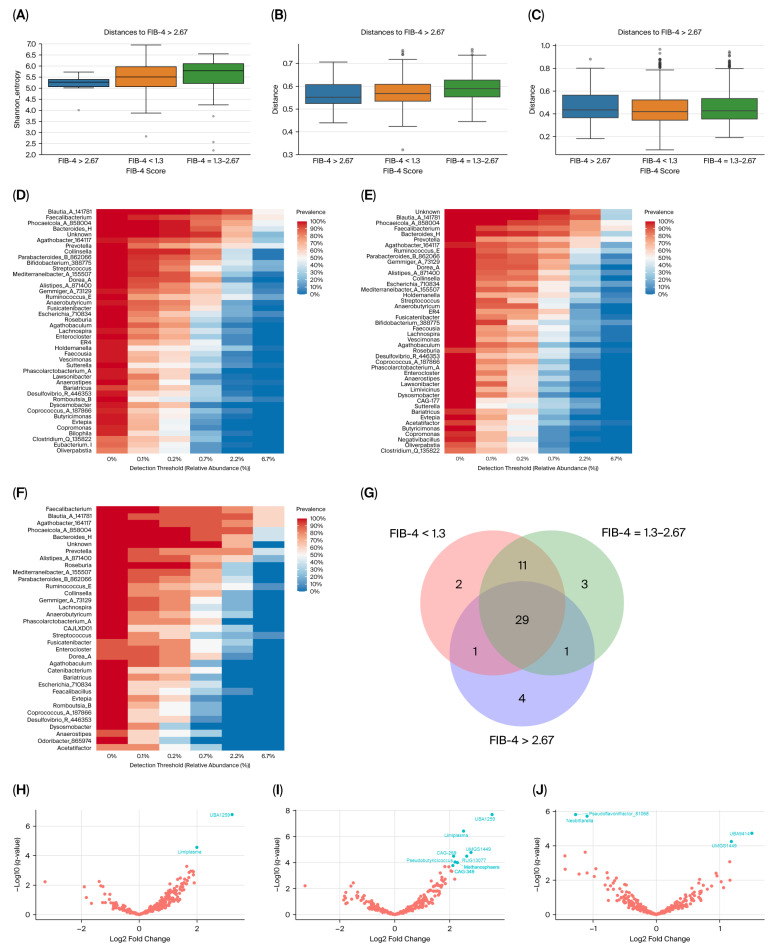
Bacterial diversity and taxa abundance differences in the gut microbiota of T2DM patients grouped by FIB-4 score. (**A**) Shannon diversity index plotted based on FIB-4 score. (**B**) Unweighted UniFrac distances (beta diversity) plotted based on FIB-4 score. (**C**) Weighted UniFrac distances (beta diversity) plotted based on FIB-4 score. (**D**) Core microbiome for patients with FIB-4 < 1.3. (**E**) Core microbiome for patients with FIB-4 = 1.3–2.67. (**F**) Core microbiome for patients with FIB-4 > 2.67. (**G**) Venn diagrams represent shared core genera between groups. (**H**) Volcano plot from ANCOMBC2 analysis at the genus level between FIB-4 > 2.67 and FIB-4 < 1.3. (**I**) Volcano plot from ANCOMBC2 analysis at the genus level between FIB-4 > 2.67 and FIB-4 = 1.3–2.67. (**J**) Volcano plot from ANCOMBC2 analysis at the genus level between FIB-4 < 1.3 and FIB-4 = 1.3–2.67. In each volcano plot, the *x*-axis (effect size) shows the log2 fold change, which represents the magnitude of the difference between the two analyzed groups. Negative values indicate features that are more abundant in the first-mentioned group in the plot legend, while positive values on the *x*-axis indicate that features are more abundant in the second-mentioned group. The *y*-axis (significance) shows the −log10 (*q* values). A larger negative log-transformed *q* value means stronger statistical significance. The threshold for significance was set as *q* < 0.05, i.e., −log10 (FDR *q* value) > 1.3. ANCOMBC2, Analysis of Composition of Microbiomes with Bias Correction 2.

Patients with a high-risk score had the lowest Shannon index when compared to those with FIB-4 < 2.67, with no significant differences between groups (*p* = 0.07; Figure 3A). Patients with FIB-4 > 2.67 showed a statistically significant separation from those of the intermediate FIB-4 scores in the unweighted Unifrac plot (*q* = 0.04; Figure 3B), but not in the weighted Unifrac plot (*q* = 0.33; Figure 3C). No differences in the beta diversity were observed between samples with intermediate and low scores (Figure 3B,C).

The core microbiota of those patients with FIB-4 < 1.3 consisted of forty-three bacterial genera (7.18% of the total; Figure 3D), whereas forty-four genera were identified as core microbiota of FIB-4 = 1.3–2.67 scores (7.68% of the total; Figure 3E), and thirty-five of those with FIB-4 > 2.67 (8.82% of the total; Figure 3F). Two bacterial genera (g__*Bilophila* and g__*Eubacterium_I*) were exclusively observed in the FIB-4 < 1.3 group (Figure 3G), three genera (g__*CAG177*, g__*Limivicinus*, and g__*Negativibacillus)* were solely observed in patients with intermediate FIB-4 values, and four genera (g__*Odoribacter_865974*, g__*Catenibacterium*, g__*Faecalibacillus*, and g__*CAJLXD01)* were present only in the FIB-4 > 2.64 group (Figure 3D–G).

ANCOMBC2 analysis revealed significantly different gut taxa abundance among patients grouped by their FIB-4 scores (Figure 3H–J and Figure S7A–D). Out of the 289 observed bacterial genera, the *UBA1259* and *Limiplasma* genera were significantly more abundant among FIB-4 < 1.3 (*q* = 4.85 × 10^−5^ and *q* = 8.11 × 10^−3^, respectively) and FIB-4 = 1.3–2.67 scores (*q* = 6.05 × 10^−6^ and *q* = 1.11 × 10^−4^, respectively) when compared to FIB-4 > 2.67 (Figure 3H–I).

#### 3.3.4. Analyses of the Gut Bacterial Metagenome According to the PNPLA3 rs738409 Genotype

**Figure 4:**
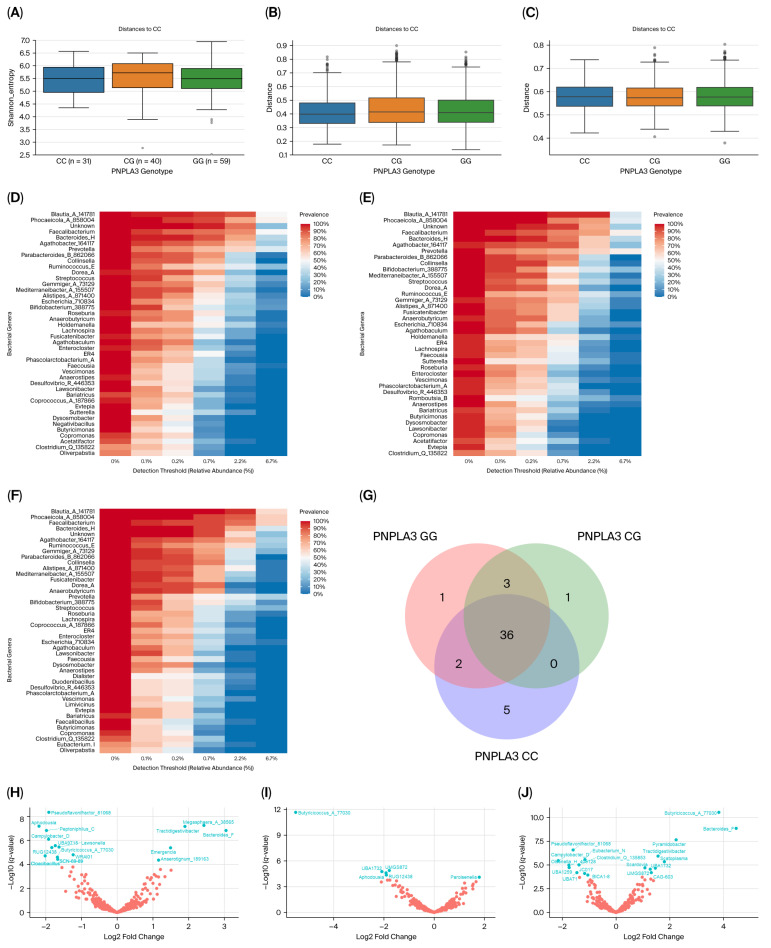
Bacterial diversity and taxa abundance differences in the gut microbiota of T2DM patients grouped by *PNPLA3* rs738409 genotype. (**A**) Shannon diversity index plotted based on PNPLA3 genotype. (**B**) Weighted UniFrac distances (beta diversity) plotted based on *PNPLA3* rs738409 genotype. (**C**) Unweighted UniFrac distances (beta diversity) plotted based on *PNPLA3* rs738409 genotype. (**D**) Core microbiome for GG carriers. (**E**) Core microbiome for heterozygous carriers. (**F**) Core microbiome for CC carriers. (**G**) Venn diagrams represent shared core genera between groups. (**H**) Volcano plot from ANCOMBC2 analysis at the genus level between CC and GG carriers. (**I**) Volcano plot from ANCOMBC2 analysis at the genus level between CC and heterozygous carriers. (**J**) Volcano plot from ANCOMBC2 analysis at the genus level between heterozygous carriers and GG carriers. In each volcano plot, the *x*-axis (effect size) shows the log2 fold change, which represents the magnitude of the difference between the two analyzed *PNPLA3* genotypes. Negative values indicate features that are more abundant in the first-mentioned genotype in the plot legend, while positive values on the *x*-axis indicate that features are more abundant in the second-mentioned genotype. The *y*-axis (significance) shows the −log10 (*q* values). A larger negative log-transformed *q* value means stronger statistical significance. The threshold for significance was set as *q* < 0.05, i.e., −log10 (FDR *q* value) > 1.3. ANCOMBC2, Analysis of Composition of Microbiomes with Bias Correction 2.

Alpha diversity analysis revealed no significant differences between genotypes (*p* = 0.57; Figure 4A). After adjusting for the geographical origin of the samples, PERMANOVA analysis showed similar weighted (*q* = 0.21; Figure 4B) and unweighted UniFrac distances (*q* = 0.42; Figure 4C) between *PNPLA3* genotypes.

The core microbiota of GG carriers consisted of forty-two bacterial genera (7.43% of the total; Figure 4D), forty genera for heterozygous carriers (7.28% of the total; Figure 4E) and forty-three genera for CC carriers (8.04% of the total; Figure 4F). The *Negativibacillus* genus was exclusively detected among the GG carriers, whereas the *Romboutsia_B* genus was characteristic of heterozygous carriers and five bacterial genera (g__*Eubacterium*_I, g__*Limivicinus*, g__*Dialister*, g__*Faecalibacillus*, *and* g__*Duodenibacillus*) were solely related to the beneficial CC genotype (Figure 4D–G).

Bacterial microbiota composition significantly differed regarding rs738409 genotype (Figure 4H–J and Figure S8A–F). Out of the 368 observed bacterial genera, *Megasphaera_A_38565* (*q* = 2.11 × 10^−5^), *Tractidigestivibacter* (*q* = 2.6 × 10^−5^), *Bacteroides_F* (*q* = 5.54 × 10^−5^), *Emergencia* (*q* = 1.48 × 10^−3^), and *Anaerotignum_189163* (*q* = 1.57 × 10^−2^) were significantly more abundant among the GG carriers, when compared with the CC genotype (Figure 4H).

## 4. Discussion

People with T2DM are at higher risk of MASLD, disease progression, and overall liver-related mortality [29]. For this reason, patients with T2DM should be prioritized for MASLD screening and risk stratification [29].

As the pathophysiology mechanisms of MASLD may differ from patient to patient with and without T2DM, studies focused on non-invasive methods for MASLD diagnosis and prognosis in the T2DM population are urgently needed. We investigated the interaction of gut microbiome signatures and genetic and clinical risk factors for MASLD in patients with T2DM from different geographical areas of Argentina.

In Argentina, MASLD affects approximately eight out of ten people with T2DM [16]. Moreover, MASLD prevalence is higher in southern Argentina (90.28%) when compared to other regions of the country (77.9–86.7%) [16]. Although we randomly recruited a cohort of 214 T2DM patients who also participated in [16], we did not observe significant regional differences in MASLD prevalence, which could be explained by the limited number of participants from some geographical areas, meaning that these areas are underrepresented in our study.

However, we detected regional variations in rates of physical activity, cholesterol levels, hypertension and cardiovascular risk. Moreover, the *PNPLA3* GG genotype, linked to Native American ancestry [17], also showed a specific distribution pattern in Argentina, which reflects the genetic background of the contemporary Argentine population after centuries of admixture [30]. Thus, the geographic, ethnic and sociocultural diversity of Argentina could reflect multiple regional determinants that influence conditions closely linked to the development of MASLD.

Gut microbiota constitute a promising source for non-invasive biomarkers in MASLD diagnosis and risk stratification [3,15]. Microbiota appear to be similar in people living within the same area and in close proximity to one another [31]. However, even within the same country, geographical and socio-economic differences may influence the human gut microbiota [31]. For this reason, national and multi-centric studies are mandatory for the identification of microbiota-derived biomarkers [3,15].

In our study, the diversity and composition of the bacterial metagenome of patients with T2DM significantly differed between the four analyzed geographical regions, with the microbiomes from SOUTH being the most distinct. These variations may be related to differences in dietary habits, cultural characteristics, and extreme climatic conditions [32]. In fact, polar weather is characteristic of the Patagonian region due to its southern location and the influence of cold air masses originating from Antarctica. Interestingly, the *Megamonas* genus, studied in Antarctic research stations [33], was present exclusively in the core microbiota from SOUTH in this study. *Megamonas* bacteria are obesity-enriched and found across diverse populations worldwide; they degrade myo-inositol, a compound involved in glycemic and lipidic metabolism [34]. These findings may suggest that, in the Patagonian region, microbe-induced obesity could demonstrate how gut microbiota dynamics shift in response to extreme environmental changes.

The rural and urban areas of Buenos Aires markedly differ in their population density (15 and 15,000 persons per km^2^, respectively, as of the 2022 census) and, despite their geographical closeness (the distance from Buenos Aires to Balcarce and Chacabuco is 416 and 212 km, respectively), we found significant differences in the gut microbiota of these populations. The metropolitan region of Buenos Aires is the most urbanized area of Argentina, and the third-largest urban agglomeration in Latin America, whereas Balcarce and Chacabuco are rural towns in the Buenos Aires province. Lifestyle and social overcrowding are important modifiers of gut microbiota diversity and composition [35]. In fact, the greater abundance of the *Succinatimonas* genus in the rural environment of Argentina was also recently reported in rural microbiomes from individuals practicing traditional lifestyles [36].

In this study, we identified MASLD-specific bacterial signatures in our T2DM cohort, such as the proinflammatory genera *Sutterella* and *Romboutsia,* which have been previously related to MASLD progression [37,38]. In addition, bacteria from the *Bilophila* genus synergize with a high-fat diet and are linked to higher glucose dysmetabolism and hepatic steatosis [39]. On the contrary, in the core microbiota of the non-MASLD group, we identified beneficial bacteria (g__*Anaerotignum*_189125, g__*Eubacterium*_I, and g__*Faecalibacillus*), which play a role in improving insulin sensitivity and intestinal gluconeogenesis [40]. This finding was expected bearing in mind first that 90.5% of the participants were receiving diabetes treatment at the time of their recruitment, and second that oral antidiabetic drugs increase short-chain fatty acid-producing bacteria, related to weight loss and anti-inflammatory effects [41]. Improving our understanding of how risk factors for advanced liver disease interact with one another is crucial in clinical practice. Previous studies, as well as the results found in our population, suggested that the *PNPLA3* GG genotype influences fibrosis progression as calculated by the FIB-4 score [42]. On the other hand, GG carriers in this population have reduced plasma lipid levels which could be explained by their reduced hepatic lipolysis and release of lipids into the bloodstream, in spite of their high liver fat content [43].

In individuals with T2DM, this genotype was also associated with better glycemic control [44]. Although this observation has raised some controversy [42], it seems that the I148M variant increases the hepatic retention of polyunsaturated fatty acids (PUFAs) and reduces their levels in triglycerides secreted by the liver both in the fasting state and postprandially [45]. PUFAs selectively suppress hepatic sterol regulatory element-binding protein (SREBP)-1 and carbohydrate response element-binding protein (ChREBP), two key transcription factors for the regulation of lipogenesis and glucose production in the liver, which are highly expressed in insulin-resistant states [46,47]. In addition, it must be taken into account that the observed relationship may be influenced by treatment allocation patterns or by region-specific differences in clinical management. Moreover, as this is a cross-sectional study, we do not know whether the better glycemic control of these patients with lower levels of HBA1c and fasting plasma glucose could be the consequence of a more careful treatment.

The fact that gut microbiota markedly differed depending on *PNPLA3* rs738409 genotype and FIB-4 score suggests that MASLD is induced in genetically predisposed subjects by multiple insults acting together [48]. In this regard, bacteria from the *Eubacterium* genus—which are relevant short-chain fatty acid producers that regulate colonic inflammation, gut barrier dysfunction and energy harvest [40]—were present among both non-MASLD patients and those patients with the lowest risk of MASLD or fibrosis progression.

On the other hand, in patients with FIB-4 > 2.67, we observed bacteria from the pro-inflammatory, pro-immunogenic, and pro-fibrogenic *Catenibacterium* genus [49]. Meanwhile, in the core microbiota of diabetic patients with MASLD and patients with the *PNPLA3* GG genotype, and in those with an intermediate risk of fibrosis (FIB-4 = 1.3–2.67), we detected the *Negativibacillus* bacterial genus. A recent study that analyzed 16S rRNA sequences from 1189 subjects concluded that *Negativibacillus* has the diagnostic potential to distinguish patients with MASLD from healthy controls and to predict MASLD progression [38]. Moreover, the presence of this bacterial genus in the human gut was correlated to reduced bile acid synthesis and increased hepatic cholesterol accumulation via the intestinal farnesoid X receptor–fibroblast growth factor 19 (FXR-FGF19) axis [50], which could be associated with the hepatic lipid retention in *PNPLA3* GG carriers. For these patients, actively consuming dietary fiber and other foods that increase short-chain fatty acids may help prevent and treat MASLD by fostering the growth of beneficial gut bacteria to the detriment of the harmful *Negativibacillus*. The association between *Negativibacillus* in GG carriers and MASLD cases described herein and reported in multi-ethnic studies [38] warrants further investigation. Thus, we used an automated stepwise forward technique and a sensitivity analysis strategy to evaluate whether adjustment variables like age, sex, BMI and FIB-4 behave as modifier variables of the aforementioned association. Results show that the FIB-4 modified the effect of the association between *Negativibacillus* and PNPLA3. The final logistic adjustment models for each stratum of the FIB-4 score showed that the association lost significance (*p* = 0.714) in the sub-group of patients with a low risk of fibrosis (FIB-4 < 1.3) but remained significant when accounting for the intermediate (*p* = 0.001) and high-risk groups (*p* = 0.004).

Our study has limitations that need to be addressed in the interest of cautious interpretation of analytical conclusions. First, due to the study design, no causal relationships could be established. Future studies are needed to determine whether the microbiome changes described herein precede or are a consequence of the development and progression of MASLD. Second, the number of samples from some geographical regions (particularly NEA and SOUTH) was limited, and the central and Andean regions of Argentina were not represented in this study. As geography is a critical confounding factor in microbiome analyses [35], this may affect the reliability of inter-regional comparisons and introduce sampling bias. Therefore, our results cannot be generalized to the entire country. Third, dietary differences could not be ruled out as a confounding factor in our study. Given the significant impact of diet on gut microbiota composition [31], the lack of dietary data from the recruited subjects substantially restricts the interpretability of these findings, especially with respect to regional variations. Additionally, a significant proportion of participants were treated with metformin and GLP-1 agonists, which are known to influence the gut microbiome [15]. This should be considered when interpreting microbial signatures associated with MASLD or the *PNPLA3* gene. Future studies should incorporate dietary assessment by food records or food frequency questionnaires. Finally, MASLD diagnosis and liver fibrosis were evaluated using abdominal ultrasound and the FIB-4 score, respectively, and not by the gold standard: liver biopsy. In addition, the FIB-4 score shows reduced accuracy in individuals scoring in the intermediate range (1.3–2.67), in older adults and in specific populations like those with diabetes. Moreover, the EASL–EASD–EASO Clinical Practice Guidelines recommend a higher lower cut-off of 2.0 for patients older than 65 years [51]. All of these methodological limitations could have led to false positives or misdiagnoses in this study. However, given the significant burden of MASLD in Latin America, local guidelines recommend ultrasonography as the initial screening tool, while FIB-4 is preferred for fibrosis risk stratification in this region [8].

## 5. Conclusions

In conclusion, despite the regional and intrinsic differences in gut microbiome, we have reported specific signatures that could be useful biomarkers of MASLD diagnosis and risk stratification in diabetic patients from Argentina. In addition, the significant interactions of gut bacterial taxa with recognized predictors for advanced liver disease in these patients could establish the basis necessary for building a potential risk prediction model.

## Figures and Tables

**Table 1:** Characteristics of the patients included in the study.

	ALL (*n* = 214)	BA City (*n* = 71)	Rural BA (*n* = 40)	NEA (*n* = 20)	NWA (*n* = 52)	SOUTH (*n* = 31)	*q* Value
Age, years, median (Q1–Q3)	61.7 (56–70)	62.5 (60–77)	63 (55–68)	63.6 (59–70)	62.9 (57–67)	59.9 (58–72)	0.07
Male gender, n (%)	110 (51.60)	37 (51.60)	22 (55)	8 (40)	32 (61.10)	19 (60)	0.2
BMI, kg/m^2^, median (Q1–Q3)	32.3 (27–34)	32.5 (29–35.75)	32.9 (30–35)	32.2 (28.75–34)	31.1 (29–34)	31.8 (26–34)	0.06
Waist circumference, cm, median (Q1–Q3)	105.5 (96–110)	106.4 (85–107)	105.7 (89.75–101)	102.8 (90.75–99)	104.3 (106.25–91.5)	103.5 (92–109)	**0.006**
Time since T2DM diagnosis, years, median (Q1–Q3)	11.1 (8.5–18.75)	12.1 (8–18)	12.6 (7–22.5)	12.9 (9–23.25)	11.1 (7–18)	8.9 (8–15)	0.1
Physical activity, n (%)	105 (48.9)	35 (50)	11 (28)	16 (82)	31 (59.4)	11 (35.5)	**0.005**
HbA1c, %, median (Q1–Q3)	7 (6–7.5)	6 (5–6.5)	7 (5.6–7.15)	6.95 (5.4–7.325)	6.5 (5.5–7.4)	8 (6–9.75)	0.4
Fasting plasma glucose, mg/dL, median (Q1–Q3)	116 (92–132.5)	119 (90–130)	116 (78.5–127.25)	126.5 (98–133.5)	115 (82–115)	114 (70–177)	0.5
Total platelets, 10^3^/µL, median (Q1–Q3)	234 (200–273.5)	243 (215.7–284.25)	212.500 (198.6–292.1)	232 (208.3–279.8)	223 (195.9–266.4)	253 (204.5–276.5)	0.38
ALT, IU/L, median (Q1–Q3)	23 (20.5–38.5)	22 (16–31.5)	22 (17.75–40.25)	25.5 (21–31.35)	24 (19–38)	30 (20–54)	0.11
AST, IU/L, median (Q1–Q3)	21 (15–26.5)	19 (17–26.5)	23 (19–32.5)	24.5 (21–28.2)	19 (18–34.5)	26 (20–39)	**0.03**
Total cholesterol, mg/dL, median (Q1–Q3)	165.5 (102.5–256)	152.5 (104.75–260)	163 (104–259.5)	175.5 (121.25–260)	165 (112–265.5)	187 (125–208)	**0.01**
Triglycerides, mg/dL, median (Q1–Q3)	136 (103–180)	112 (100–149)	142 (105–185)	166.5 (108–228.5)	135 (109–185)	166 (110–187)	0.48
Hypertension, n (%)	147 (68.90)	53 (74.20)	31 (77.50)	16 (80)	25 (48.60)	19 (61.30)	**0.03**
Cardiovascular risk, high to critic, n (%)	133 (62.10)	53 (74.20)	38 (95)	19 (95)	39 (75.70)	22 (71)	**0.02**
PNPLA3, GG genotype, n (%) *	95 (50)	25 (40.30)	18 (45)	12 (60)	24 (64.9)	16 (51.6)	0.14
PNPLA3, CG genotype, n (%) *	62 (32.6)	24 (38.7)	12 (30)	6 (30)	11 (29.7)	9 (29.05)	0.82
PNPLA3, CC genotype, n (%) *	33 (17.4)	13 (21)	10 (25)	2 (10)	2 (5.4)	6 (19.35)	0.15
PNPLA3, G allele, n (frequency) *	252 (0.66)	74 (0.6)	48 (0.6)	30 (0.75)	59 (0.8)	41 (0.66)	**0.02**
PNPLA3, C allele, n (frequency) *	128 (0.34)	50 (0.4)	32 (0.4)	10 (0.25)	15 (0.2)	21 (0.44)	
Diagnosis of MASLD, n (%)	167 (77.90)	53 (74.20)	32 (80)	17 (85)	40 (76)	26 (83.90)	0.75
FIB-4 score > 1.3, n (%)	96 (44.7)	23 (32.3)	26 (65)	11 (55)	21 (40.5)	11 (35.5)	**0.01**

BA city: Buenos Aires city; rural BA: rural Buenos Aires; NEA: northeastern Argentina; NWA: northwestern Argentina; SOUTH: southern region or Patagonia; BMI: body mass index; T2DM: type 2 diabetes mellitus; HbA1c: glycated hemoglobin; ALT: alanine aminotransferase; IU: international units; AST: aspartate aminotransferase; MASLD: metabolic dysfunction-associated steatotic liver disease; PNPLA3: patatin-like phospholipase domain-containing 3. Statistically significant *p* values are in bold. * The *PNPLA3* gene was successfully amplified in 190 samples (62 from BA city, 40 from rural BA, 37 from NWA, 20 from NEA, and 31 from SOUTH).

**Table 2:** Univariate and multivariate logistic regression analyses to identify independent factors associated with the *PNPLA3* rs738409 genotype.

Patient Characteristics	*PNPLA3* GG Genotype (*n* = 95)	*PNPLA3* CC/CG Genotype (*n* = 95)	Univariate Analysis	Multivariate Analysis		
			*p* Value	B	OR (95% CI)	*p* Value
Total platelets, 10^3^/µL, median (Q1–Q3)	225 (195.9–268.7)	224 (186–264)	0.2			
Fasting plasma glucose, mg/dL, median (Q1–Q3)	105 (71–125)	111 (82–136)	**0.002**	−1	0.73 (0.87–0.98)	**0.008**
HbA1c, %, median (Q1–Q3)	6 (5–7.8)	6.3 (5.5–9)	**0.002**	−4.8	0.875 (0.27–0.91)	**0.004**
ALT, IU/L, median (Q1–Q3)	18 (11–29)	21 (15–30)	0.3			
AST, IU/L, median (Q1–Q3)	19 (15–21.5)	23 (18–25)	0.3			
Total cholesterol, mg/dL, median (Q1–Q3)	188 (110–225)	193 (105–250)	**0.01**	−1.4	0.525 (0.30–0.74)	**0.02**
Triglycerides, mg/dL, median (Q1–Q3)	155 (102–179)	159 (109–183)	0.5			
Time since T2DM diagnosis, years, median (Q1–Q3)	11.5 (9–22)	12 (8–19)	0.09			
Hypertension, *n* (%)	75 (78.9)	80 (84.2)	0.45			
Diagnosis of MASLD, *n* (%)	79 (83.2)	65 (68.4)	**0.03**	2.6	1.3 (0.78–1.3)	0.85
FIB-4 score, median (Q1–Q3)	2.1 (1.2–3.4)	1.1 (0.5–2.6)	**0.0001**	9.4	2.06 (4.37–16.48)	**0.0008**

Statistically significant *p* values are in bold. Multivariate logistic regression analysis was adjusted by age, gender and BMI. OR: odds ratio; 95% CI: 95% confidence interval.

## Data Availability

Raw sequences of 16S rRNA gene reported herein have been deposited in NCBI database (http://www.ncbi.nlm.nih.gov/bioproject/1291719, accessed on 29 December 2025).

## Supplementary Materials

The following supporting information can be downloaded at: https://www.mdpi.com/article/10.3390/life16020283/s1: Figure S1: Map showing the location of the recruitment centers in the different geographical regions of Argentina. The size of the data point is proportional to the number of recruited patients at each center. The same colors are used to show each geographical region on the map. Figure S2: STROBE participant flow chart; Figure S3: Association of the genotype of the rs738409 polymorphism in the *PNPLA3* gene with FIB-4 score (A), fasting blood glucose levels (B), HbA1c (C), and serum total cholesterol concentrations (D) in recruited patients. Mean values and standard deviations, and *p* values from univariate analyses are shown. HbA1c: glycated hemoglobin; SNP: single-nucleotide polymorphism; PNPLA3: Patatin-like phospholipase domain-containing 3. Figure S4: Rarefaction plot. The number of sequences sampled is shown on the *x*-axis while the y-axis depicts the estimated number of observed features. The red vertical dotted line marks the rarefaction depth chosen. Figure S5: Differential taxa abundance at the family and species levels of the gut microbiota of T2DM patients from various geographical regions in Argentina. (A) Volcano plot from ANCOMBC2 analysis at the family level between BA city and rural BA. (B) Volcano plot from ANCOMBC2 analysis at the family level between NWA and SOUTH. (C) Volcano plot from ANCOMBC2 analysis at the family level between BA city and SOUTH. (D) Volcano plot from ANCOMBC2 analysis at the family level between rural BA and SOUTH. (E) Volcano plot from ANCOMBC2 analysis at the family level between NWA and rural BA. (F) Volcano plot from ANCOMBC2 analysis at the species level between BA city and rural BA. (G) Volcano plot from ANCOMBC2 analysis at the species level between NWA and SOUTH. (H) Volcano plot from ANCOMBC2 analysis at the species level between BA city and SOUTH. (I) Volcano plot from ANCOMBC2 analysis at the species level between rural BA and SOUTH. (J) Volcano plot from ANCOMBC2 analysis at the species level between NWA and rural BA. (K) Volcano plot from ANCOMBC2 analysis at the species level between BA city and NWA. In each volcano plot, the *x*-axis (effect size) shows the log2 fold change, which represents the magnitude of the difference between the two analyzed geographical regions. Negative values indicate features that are more abundant in the first- mentioned region in the plot legend, while positive values on the *x*-axis indicate that features are more abundant in the second-mentioned geographical region. The *y*-axis (significance) shows the −log10 (*q* values). A larger negative log-transformed *q* value means stronger statistical significance. The threshold for significance was set as *q* < 0.05, i.e., −log10 (FDR *q* value) > 1.3. No differential abundant taxa were detected at the family level between BA city and NWA. ANCOMBC2, Analysis of Composition of Microbiomes with Bias Correction 2. Figure S6: Differential taxa abundance at the family and species levels of the gut microbiota of T2DM patients with or without MASLD. (A) Volcano plot from ANCOMBC2 analysis at the family level between non-MASLD and MASLD samples. (B) Volcano plot from ANCOMBC2 analysis at the species level between non-MASLD and MASLD samples. The *x*-axis (effect size) shows the log2 fold change, which represents the magnitude of the difference between the two analyzed groups. Negative values indicate features that are more abundant in the non-MASLD group, while positive values on the *x*-axis indicate that features are more abundant in the MASLD group. The *y*-axis (significance) shows the −log10 (*q* values). A larger negative log-transformed *q* value means stronger statistical significance. The threshold for significance was set as *q* < 0.05, i.e., −log10 (FDR *q* value) > 1.3. ANCOMBC2, Analysis of Composition of Microbiomes with Bias Correction 2. Figure S7: Differential taxa abundance at the family and species levels of the gut microbiota of T2DM patients grouped by FIB-4 score. (A) Volcano plot from ANCOMBC2 analysis at the family level between FIB-4 > 2.67 and FIB-4 = 1.3–2.67. (B) Volcano plot from ANCOMBC2 analysis at the species level between FIB-4 > 2.67 and FIB-4 < 1.3. (C) Volcano plot from ANCOMBC2 analysis at the species level between FIB-4 > 2.67 and FIB-4 = 1.3–2.67. (D) Volcano plot from ANCOMBC2 analysis at the species level between FIB-4 < 1.3 and FIB-4 = 1.3–2.67. In each volcano plot, the *x*-axis (effect size) shows the log2 fold change, which represents the magnitude of the difference between the two analyzed groups. Negative values indicate features that are more abundant in the first-mentioned group in the plot legend, while positive values on the *x*-axis indicate that features are more abundant in the second-mentioned group. The *y*-axis (significance) shows the −log10 (*q* values). A larger negative log-transformed *q* value means stronger statistical significance. The threshold for significance was set as *q* < 0.05, i.e., −log10 (FDR *q* value) > 1.3. ANCOMBC2, Analysis of Composition of Microbiomes with Bias Correction 2. Figure S8: Differential taxa abundance at the family and species levels of the gut microbiota of T2DM patients grouped by *PNPLA3* rs738409 genotype. (A) Volcano plot from ANCOMBC2 analysis at the family level between CC and GG carriers. (B) Volcano plot from ANCOMBC2 analysis at the family level between CC and heterozygous carriers. (C) Volcano plot from ANCOMBC2 analysis at the family level between heterozygous and GG carriers. (D) Volcano plot from ANCOMBC2 analysis at the species level between CC and GG carriers. (E) Volcano plot from ANCOMBC2 analysis at the species level between CC and heterozygous carriers. (F) Volcano plot from ANCOMBC2 analysis at the species level between heterozygous and GG carriers. In each volcano plot, the *x*-axis (effect size) shows the log2 fold change, which represents the magnitude of the difference between the two analyzed *PNPLA3* rs738409 genotypes. Negative values indicate features that are more abundant in the first-mentioned genotype in the plot legend, while positive values on the *x*-axis indicate that features are more abundant in the second-mentioned genotype. The *y*-axis (significance) shows the −log10 (*q* values). A larger negative log-transformed *q* value means stronger statistical significance. The threshold for significance was set as *q* < 0.05, i.e., −log10 (FDR *q* value) > 1.3. ANCOMBC2, Analysis of Composition of Microbiomes with Bias Correction 2.
