# Supplementary figures and images for: Interactions Between the Gut Microbiome and Genetic and Clinical Risk Factors for Metabolic Dysfunction-Associated Steatotic Liver Disease (MASLD) in Patients with Type 2 Diabetes Mellitus from Different Geographical Regions of Argentina
Source: Life (Basel). 2026 Feb 6;16(2):283. doi: 10.3390/life16020283 (PMC12942294; doi:10.3390/life16020283)

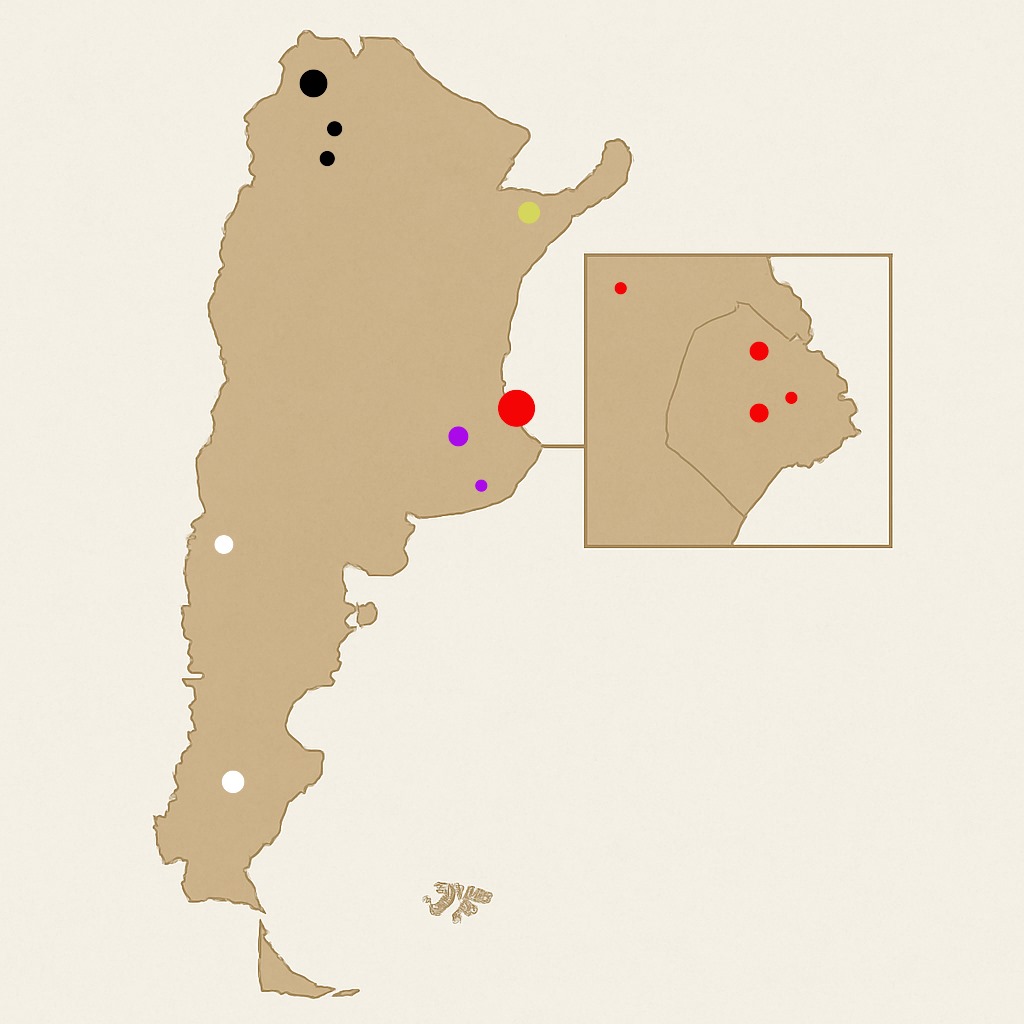

Supplement: Supplementary file 1 [file life-16-00283-s001.zip › Figure S1.jpeg]

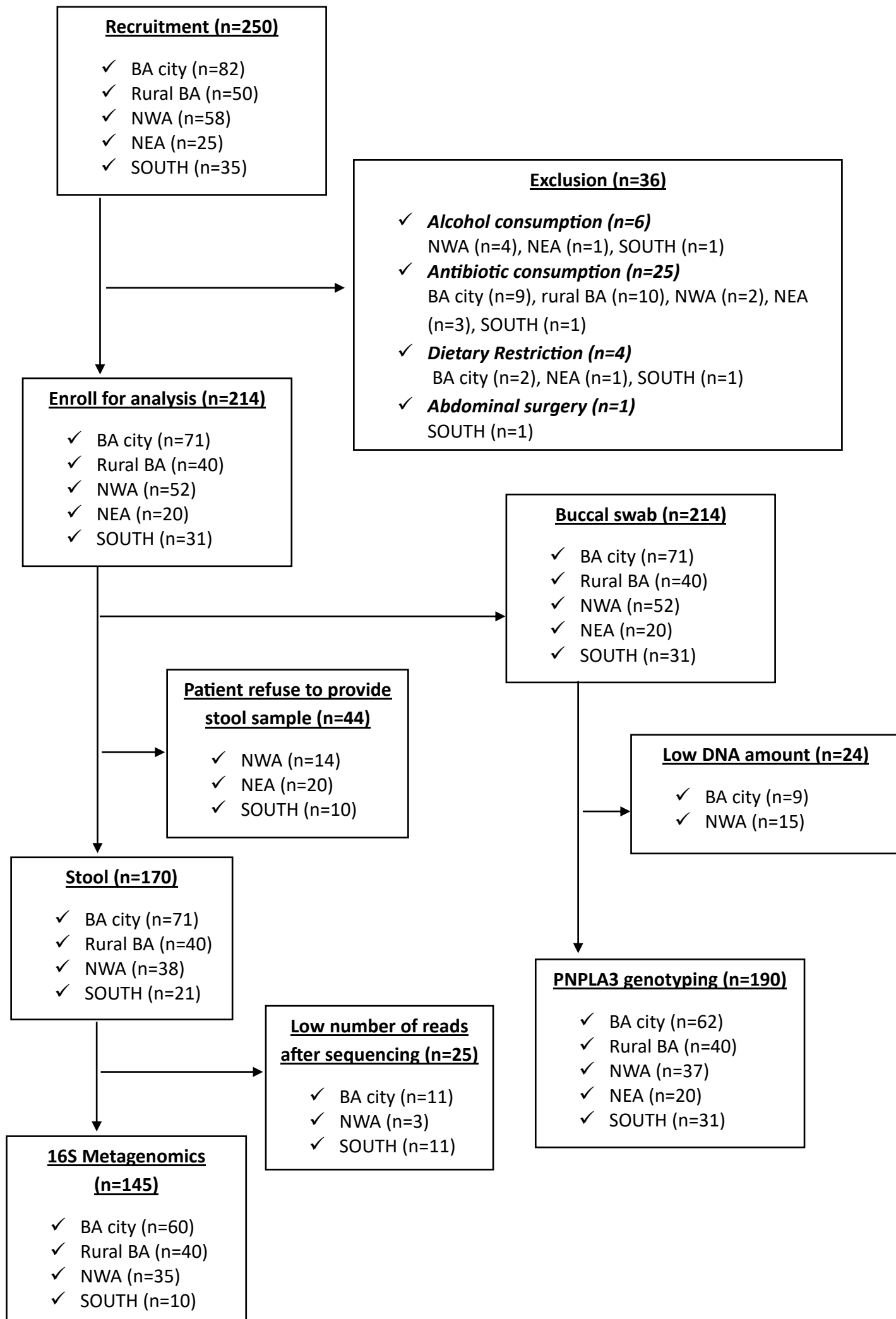

Supplement: Supplementary file 1 [file life-16-00283-s001.zip › Figure S2.pdf]

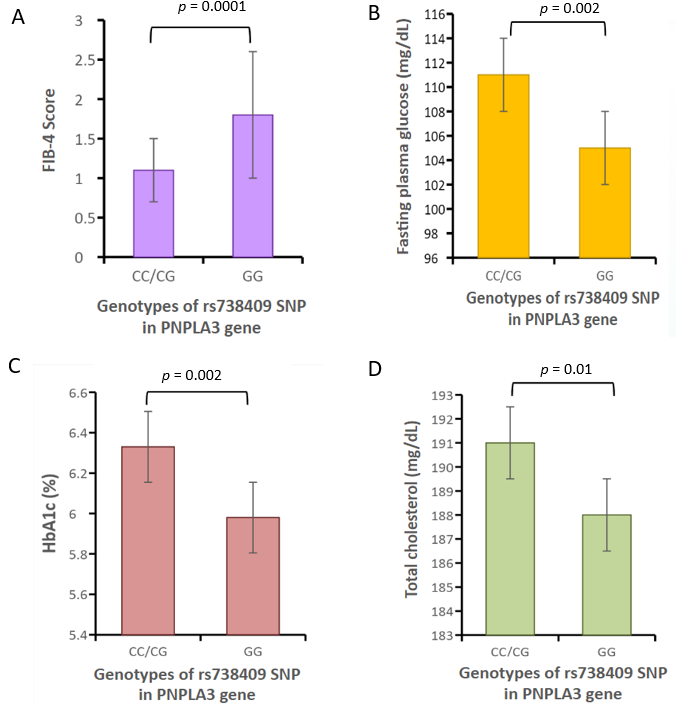

Supplement: Supplementary file 1 [file life-16-00283-s001.zip › Figure S3.png]

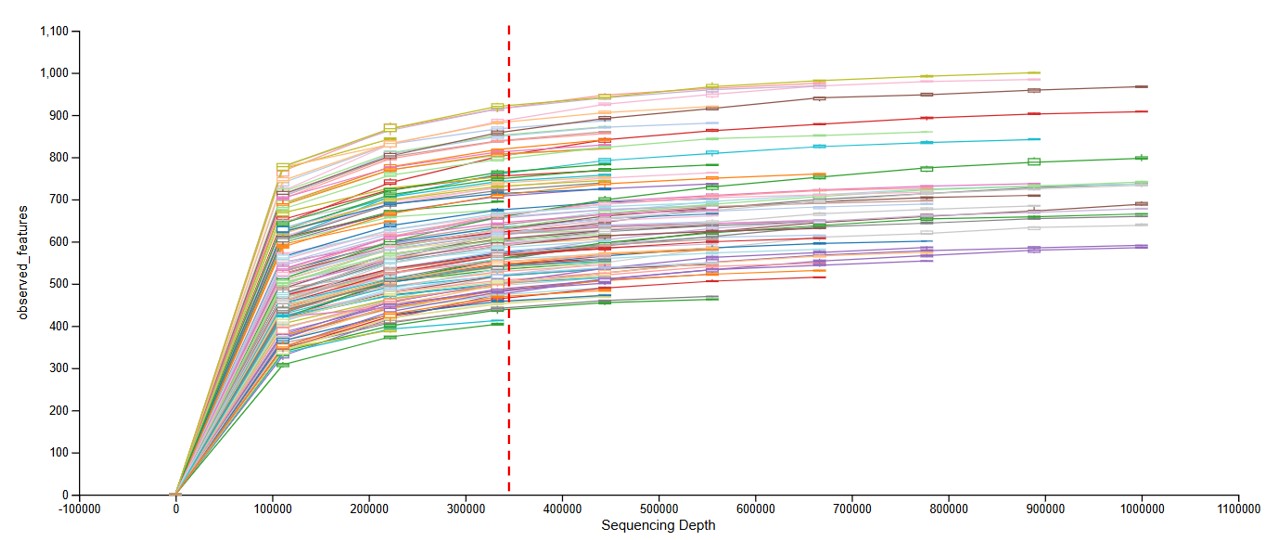

Supplement: Supplementary file 1 [file life-16-00283-s001.zip › Figure S4.jpg]

## Slide 1
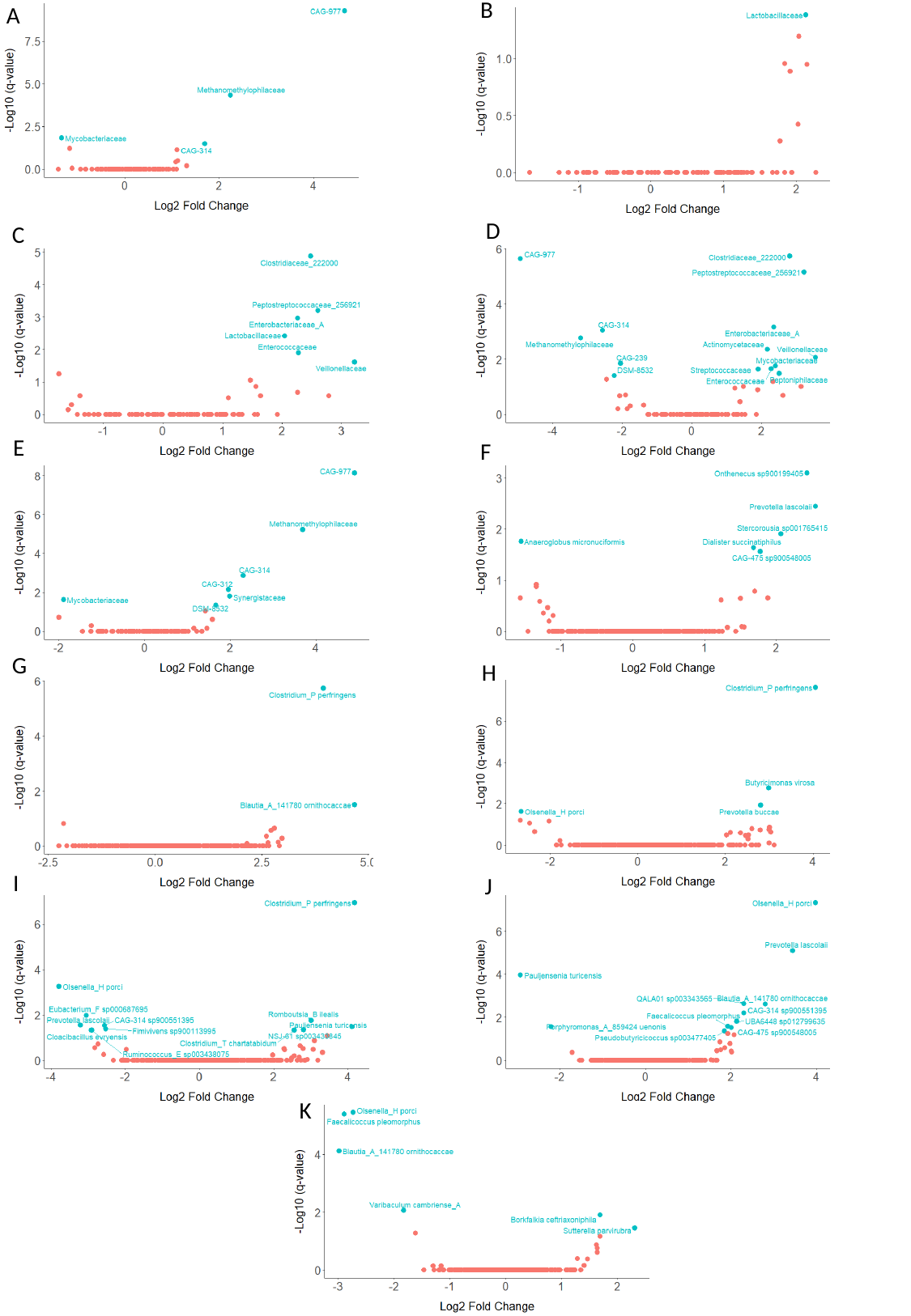

B
A
D
C
E
F
G
H
I
J
K

Supplement: Supplementary file 1 [file life-16-00283-s001.zip › Figure S5.pptx]

## Slide 1
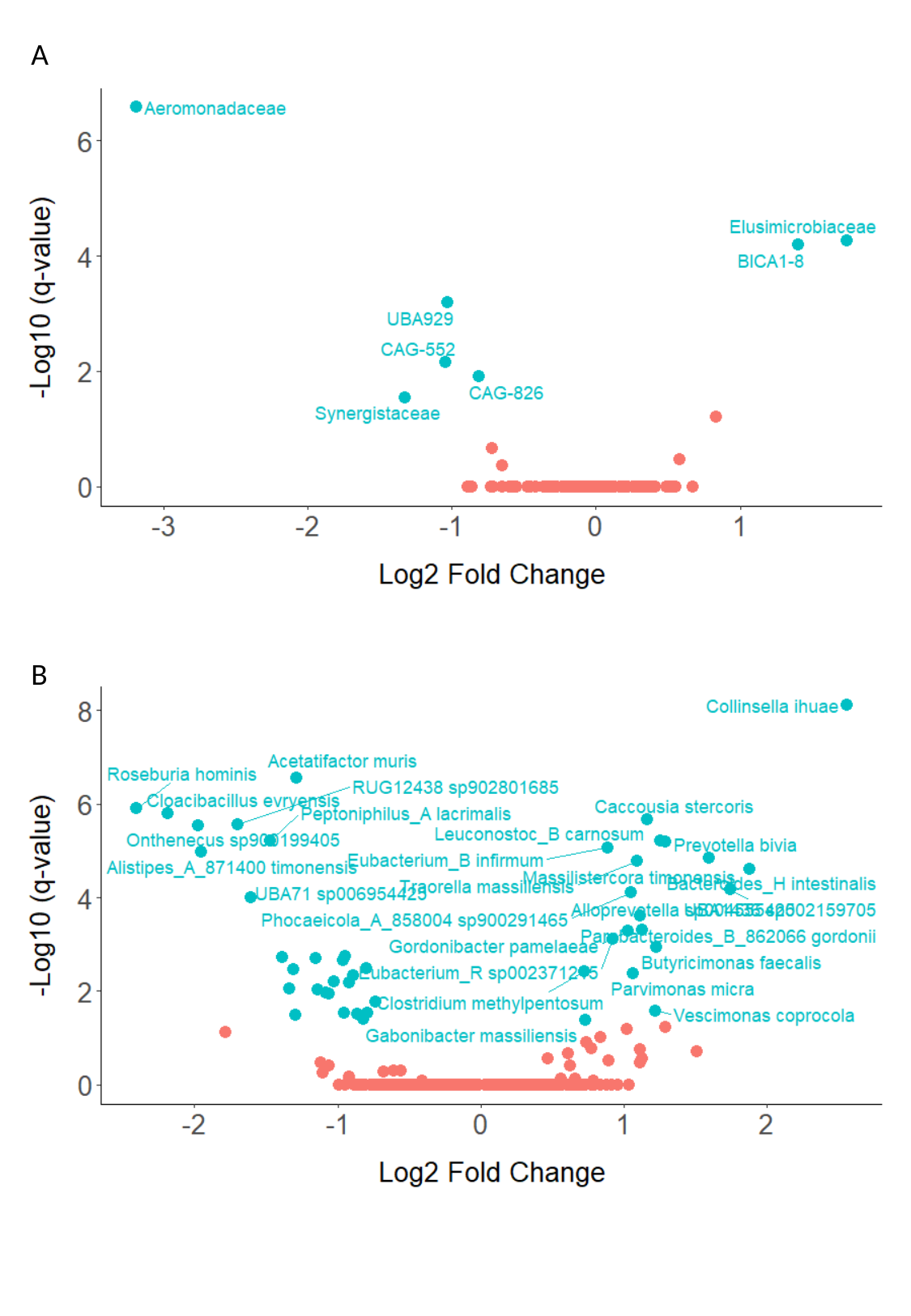

A
B

Supplement: Supplementary file 1 [file life-16-00283-s001.zip › Figure S6.pptx]

## Slide 1
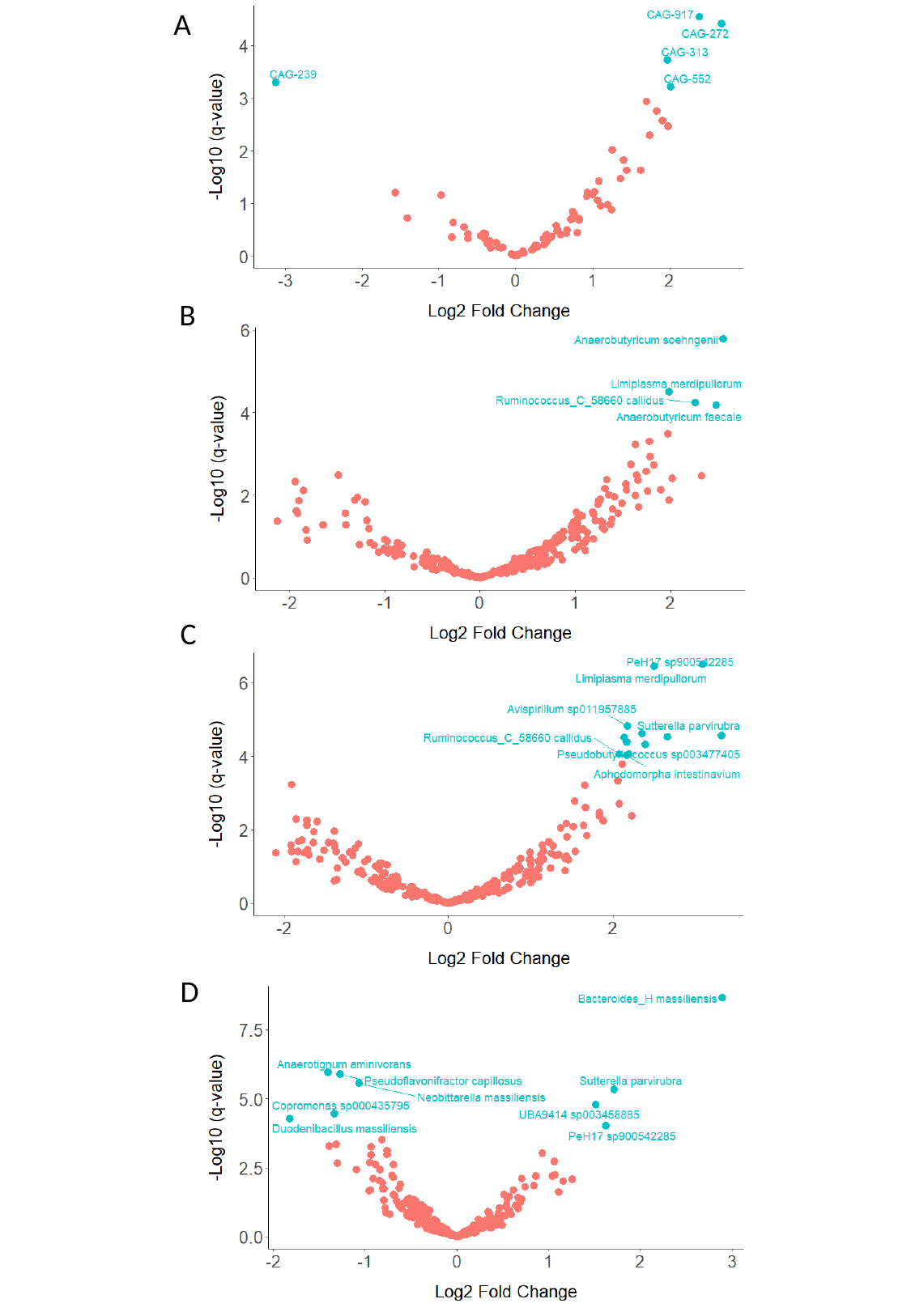

A
B
C
D

Supplement: Supplementary file 1 [file life-16-00283-s001.zip › Figure S7.pptx]

## Slide 1
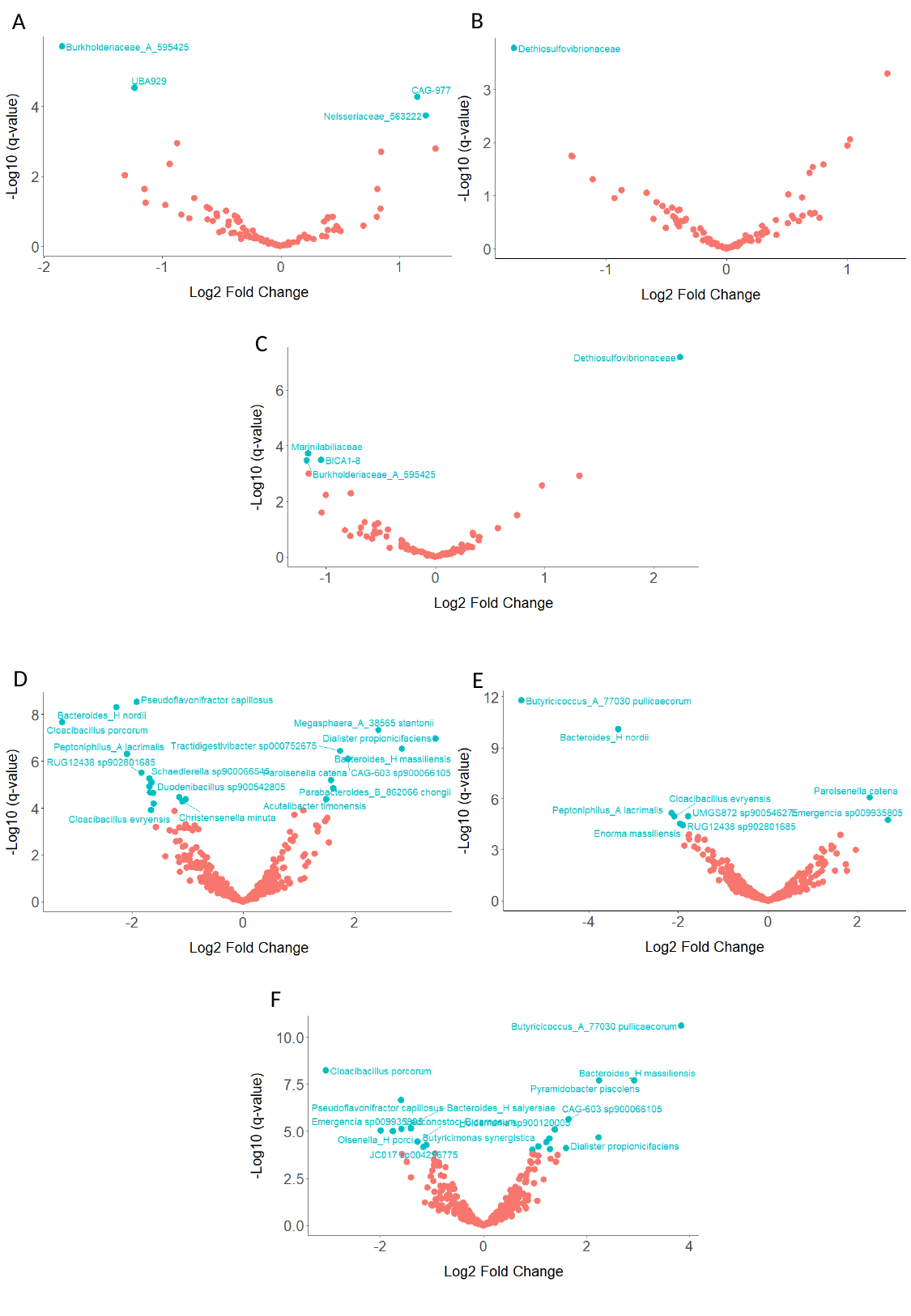

A
B
C
D
E
F

Supplement: Supplementary file 1 [file life-16-00283-s001.zip › Figure S8.pptx]
